# Influence of sports on cortical excitability in patients with spinal cord injury: a TMS study

**DOI:** 10.3389/fmedt.2024.1297552

**Published:** 2024-05-15

**Authors:** Vanessa N. Frey, Patrick B. Langthaler, Nora Renz, Georg Zimmermann, Christopher Höhn, Kerstin Schwenker, Aljoscha Thomschewski, Alexander B. Kunz, Yvonne Höller, Raffaele Nardone, Eugen Trinka

**Affiliations:** ^1^ Department of Neurology, Neurointensive Care and Neurorehabilitation, Member of the European Reference Network EpiCARE, Christian Doppler University Hospital, Centre for Cognitive Neuroscience, Paracelsus Medical University Salzburg, Salzburg, Austria; ^2^ Spinal Cord Injury and Tissue Regeneration Center, Paracelsus Medical University, Salzburg, Austria; ^3^ Department of Mathematics, Paris Lodron University, Salzburg, Austria; ^4^ IDA Lab Salzburg, Team Biostatistics and Big Medical Data, Paracelsus Medical University Salzburg, Salzburg, Austria; ^5^ Laboratory for Sleep, Cognition and Consciousness Research, Department of Psychology, Centre for Cognitive Neuroscience, University of Salzburg, Salzburg, Austria; ^6^ Karl Landsteiner Institute for Neurorehabilitation and Space Neurology Salzburg, Salzburg, Austria; ^7^ Faculty of Psychology, University of Akureyri, Akureyri, Iceland; ^8^ Department of Neurology, Tappeiner Hospital, Meran, Italy

**Keywords:** movement preparation, paraplegia, physical activity, transcranial magnetic stimulation, cortical excitability

## Abstract

**Background:**

Patients with spinal cord injury (SCI) show abnormal cortical excitability that might be caused by deafferentation. We hypothesize a reduced short-interval intracortical inhibition preceding movement in patients with SCI compared with healthy participants. In addition, we expect that neuroplasticity induced by different types of sports can modulate intracortical inhibition during movement preparation in patients with SCI.

**Methods:**

We used a reaction test and paired-pulse transcranial magnetic stimulation to record cortical excitability, assessed by measuring amplitudes of motor-evoked potentials in preparation of movement. The participants were grouped as patients with SCI practicing wheelchair dancing (*n* = 7), other sports (*n* = 6), no sports (*n* = 9), and healthy controls (*n* = 24).

**Results:**

There were neither significant differences between healthy participants and the patients nor between the different patient groups. A non-significant trend (*p* = .238), showed that patients engaged in sports have a stronger increase in cortical excitability compared with patients of the non-sportive group, while the patients in the other sports group expressed the highest increase in cortical excitability.

**Conclusion:**

The small sample sizes limit the statistical power of the study, but the trending effect warrants further investigation of different sports on the neuroplasticity in patients with SCI. It is not clear how neuroplastic changes impact the sensorimotor output of the affected extremities in a patient. This needs to be followed up in further studies with a greater sample size.

## 1 Introduction

The ability of the brain to adapt to new physiological as well as pathological circumstances is of high relevance to sustain functionality and ensure survival. When the spinal cord gets severely injured, the brain is deafferented from sensory input and adopts to this condition with neuroplastic changes (1). Especially cortical structures are highly plastic and adaptable as observed in animal models and studies in humans with deafferentation (2–7). One trigger for these neuroplastic changes is cortical excitability, which is increased by long-term potentiation and decreased by long-term depression (8). It can be quantitatively measured *in vivo* by motor-evoked potentials (MEP). MEPs are produced from brief, indirectly induced descending action potentials originated by the activation of pyramidal neurons (9). This is activated by a combination of intrinsic characteristics of the corticospinal tract and inhibitory–excitatory circuits depending on the timing, the intensity, and synchronicity of the stimulus and the direction of the induced current (10, 11). Transcranial magnetic stimulation (TMS) paired pulses (consisting of a sub-threshold conditioning stimulus followed by a supra-threshold test stimulus) with a short interstimulus interval (ISI) of 3 ms provoke short-interval intracortical inhibition (SICI). This results in a reduced MEP amplitude due to a summation at the pyramidal neurons, of presynaptic low-threshold γ-aminobutyric acid (GABA)ergic activity elicited by the sub-threshold pulse and the high-threshold non-N-Methyl-D-aspartic acid (NMDA) activity elicited by the supra-threshold pulse (12–14). In patients with spinal cord injury (SCI), a reduced inhibition of excitability was observed previously by our and other research groups (15–18). Remarkably, this effect was documented as early as within the first few days after injury (19). Decreased inhibition is also observed in the spinal cord of patients with SCI (20, 21), which might be the cause for the typical symptoms of uncontrolled muscle contractions and spasticity (17). Another well-known trigger of plastic changes in the brain is exercise. There are several experimental animal studies demonstrating the influence of physical training on structural changes in the brain. Graziano et al. demonstrated that neurons in the cortex representing the deafferented hind limb in rats increased their sensitivity to tactile stimuli applied to the forelimb after passive bike exercise compared with animals who did not exercise (22). de Leon et al. used weight-supported treadmill training in mid-thoracic transected rats and showed that even small amounts of training caused functional improvement of gait and increased the immune reactivity of the brain-derived neurotrophic factor (BDNF) (23). BDNF is a modulator of neuroplasticity and plays an important role in increasing long-term potentiation and long-term depression (24–26). In athletes with SCI, six times higher BDNF levels were found at rest compared with BDNF levels in healthy participants (27). In the same study it was found that after 10 min of light handbike training, the basic BDNF level increased by 1.5 times. Therefore, the authors suggested that exercise might enhance neuroplasticity in patients with SCI. In addition, previous work from our group revealed increased cortical excitability in patients with SCI after passive and active cycling (28). A study on healthy expert tennis players gave evidence for a specific effect by type of sport on plastic changes in the brain (29). Specifically, in these experienced tennis players the cortical excitability, as tested via single-pulse TMS, increased after imagining playing tennis, yet not after imagining playing golf or table tennis. Another study revealed that cortical excitability in healthy professional racquet players was increased in the cortex representing the hand muscles and laterally and medially shifted on the motor cortex, compared with non-professional racquet players (30). In healthy individuals, SICI is enhanced during rest, after a no-go signal (i.e., inhibition of movement), and during movement in muscles that are not involved in the action (31, 32). SICI is steadily reduced in preparation of a movement to increase the cortical excitability necessary for conducting a subsequent action (32–35). As mentioned previously, the cortical excitatory balance is disturbed in patients with SCI, resulting in a different pattern of SICI than in healthy participants (9, 36, 37).

Based on this prior research, we hypothesize that there is a sports-specific neuroplasticity in patients with SCI. We expect a reduced SICI preceding movement in patients compared with healthy participants, which might be influenced by inhibited afferent sensory input (38). To test this hypothesis we measured cortical excitability in preparation of a movement in a group of patients with SCI that regularly conduct wheelchair dancing, in a group that practiced other sports including marathon driving, hand-biking, and basketball, and in a non-sportive group of patients with SCI. Patients practicing wheelchair dancing spend a lot of time on imagination of movement (i.e., preparing, memorizing, and practicing a choreography) considering the complexity of movements that differ in nature, speed, and order. By contrast, patients who practice the other sports, especially marathon drivers and hand-bikers, are used to rather monotonous movements and focus on speed and endurance. The patient groups were compared with sportive and non-sportive healthy participants as we assume that according to the mentioned studies (29, 30) cortical excitability also differs between these groups. If the characteristics of the single sports influence the measured correlate of neuroplasticity in different ways, it is important to study the relevant consequences of the sport a patient is practicing on the outcome of the disease. Investigating these effects might give new inputs for therapeutic approaches, especially on an individual level.

## 2 Materials and methods

### 2.1 Participants

We recruited 25 patients with SCI from the Department of Neurology, Neurointensive Care, and Neurorehabilitation, of the Christian Doppler University Hospital, Salzburg, Austria, and by contacting wheelchair sports clubs. Patients were only included in the study if they did not have craniocerebral injuries in the past or suffered from other neurological diseases. Three patients had to be excluded from MEP analysis as either no motor potential could be evoked or only in some conditions. The clinical characteristics of the included patients can be found in Table 1. Patients were categorized into three groups: dancers, who were recruited from the local Wheelchair Dancer Clubs in two towns (Salzburg and Linz, Austria) (*n* = 7); patients conducting other sports (*n* = 6); and non-sportive patients (*n* = 9).

**Table 1:** Overview of individual patients with spinal cord injury including age, lesion characteristics, ASIA score, and sport they practice.

	Gender	Age	T/NT	ASIA score	Lesion height	C/IC	Age injury	Sport
1	W	62	NT	B	Th10	C	5 months	Non
2	W	47	T	A	Th12	C	4 months	Non
3	M	64	NT	C	C5	IC	20 years	Non
4	W	46	T	C	L1	IC	36 years	Non
5	W	31	T	A	Th5	C	5 years	Non
6	M	20	NT	A	Th10	C	2 months	Non
7	M	60	T	D	C4	IC	5 years	Non
8	M	49	T	D	C6	IC	4 years	Non
9	M	57	T	A	Th12	C	13 years	Non
10	W	45	T	B	C7	IC	7 years	WCD
11	W	48	T	C	L1	IC	28 years	WCD
12	M	70	T	D	L2	IC	5 months	WCD
13	W	61	T	A	Th8	C	6 years	WCD
14	W	52	T	A	Th12	C	36 years	WCD
15	W	32	T	A	Th7	C	17 years	WCD
16	M	42	T	A	Th9	C	25 years	WCD
17	M	47	T	A	Th5	C	25 years	Marathon
18	M	51	T	A	L2	C	33 years	Handbike
19	M	66	NT	C	L3	IC	7 years	Handbike
20	M	55	NT	A	L3	C	31 years	Handbike
21	W	40	T	B	L2	IC	18 years	Basketball
22	M	30	T	A	L5	C	10 years	Basketball

T, traumatic injury; NT, non-traumatic injury; C, complete SCI; IC, incomplete SCI; C*x*, cervical; Th*x*, thoracic; L*x*, lumbar; WCD, wheelchair dancing; Non, not conducting sports regularly.

Patients were included in the sportive group if they exercised more than once a week. Altogether 30 healthy participants were recruited, of which 6 had to be excluded from the MEP analysis as the data could not be processed sufficiently (too many artifacts or indefinable amplitudes). Among the healthy participants, 50% conducted different and multiple kinds of sports. Most common sports reported were running, weight training, and bicycling (mountain biking, as well as road bicycling). The other 50% of healthy participants were non-sportive.

The study was approved by the local ethics committee (415-E/1890/11-2016) and all participants signed an informed consent form. The methods were tolerated well by participants and no adverse or unexpected events occurred.

### 2.2 Reaction test and TMS

The participants conducted a reaction test on the computer (Psychomotor Vigilance Test). A black background with a yellow rectangle was visible for 6–10 s (duration was randomized within this range), until it changed its color to red. The participants were advised to perform a mouse click as soon as the rectangle changed color. This was performed 10 times for practice and 10 times to determine the mean reaction time (RT) of each participant. Only reaction times that were faster than the low pass (500 ms) were registered, while slower reaction times were disregarded. The paradigm continued until 10 trials were below the low pass mark. A paradigm prepared with Presentation® (Neurobehavioral Systems) was used to display the test and to collect data about the individual RT.

To test cortical excitability, the TMS paired pulses were applied on the hand area of the left primary motor cortex (M1) via a figure-of-eight coil. First, the individual resting motor threshold (RMT) and the hot spot of the first dorsal interosseous (FDI) were determined via single TMS pulses according to the procedure described by Rossini et al. (39). For the paired pulses, we used an ISI of 3 ms with an intensity of 80% of RMT for the first stimulus—the conditioning stimulus, and 120% for the second stimulus, the test stimulus. The two TMS devices by Mag and More and the BiStim^2^ module (Magstim Co., Whitland Dyfed, UK) were triggered by the program Presentation®, activating the pulses (two TMS devices are necessary to produce two stimuli with such a short interstimulus interval). To record MEPs, bipolar electromyography (EMG) electrodes were fixed at the FDI of the right hand and the four-channel EMG System Tru Trace (Dr. Langer Diagnostics, München Germany) was used to measure MEPs.

### 2.3 Study design

**Figure 1:**
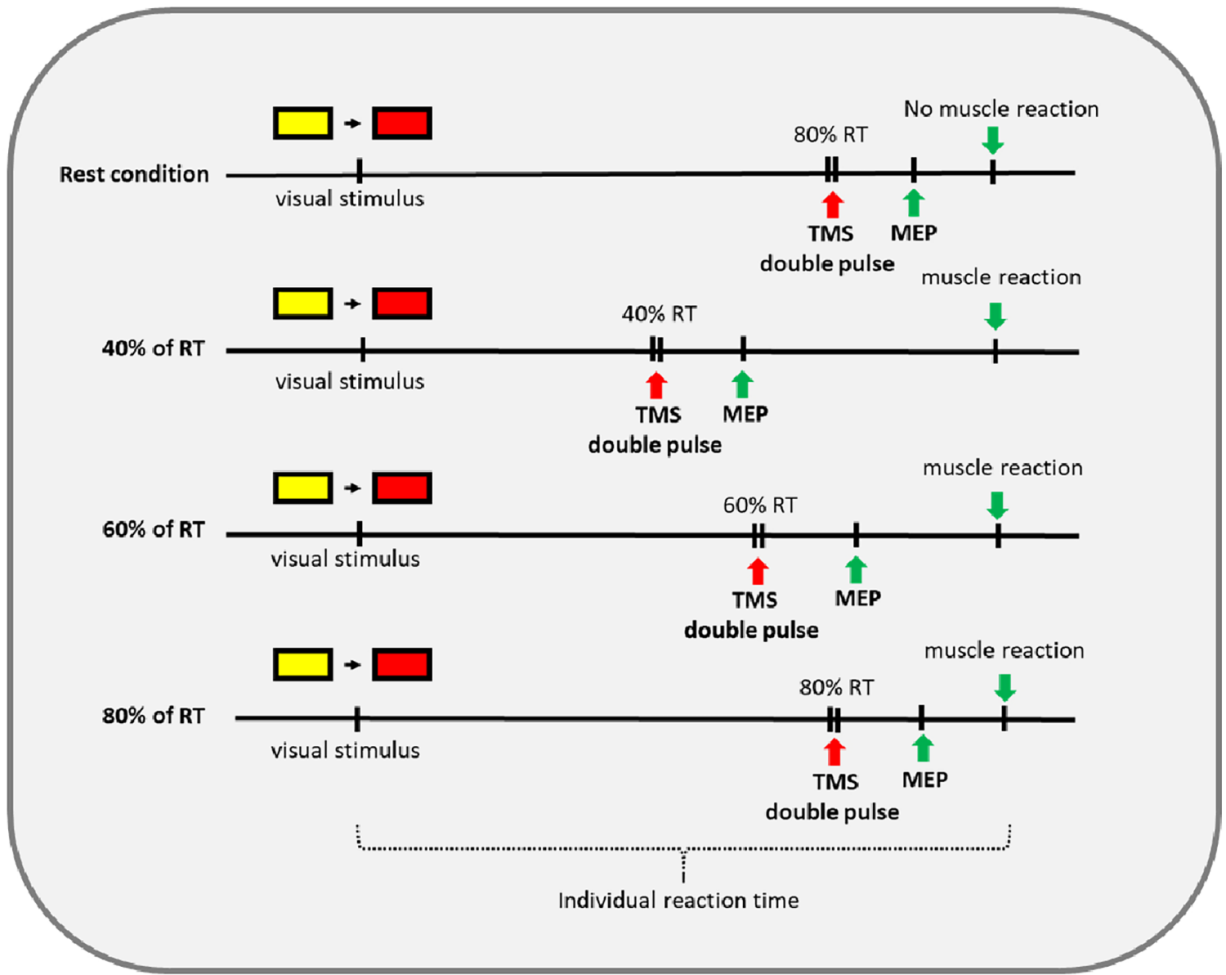
Timeline of the four different conditions rest, 40%, 60%, and 80% of RT.

**Figure 2:**
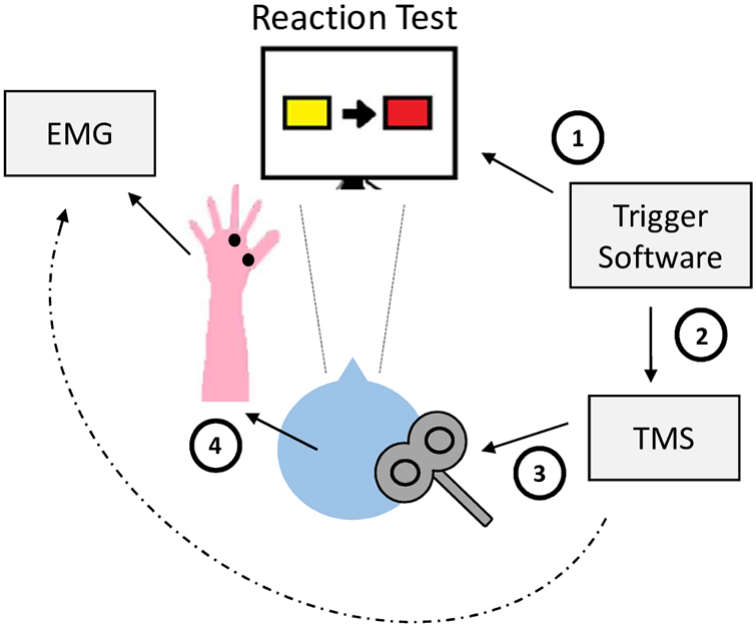
Experimental setup. (1) Stimulus from software inducing change of rectangle color. (2) Trigger from software initiating TMS pulses. (3) TMS pulses applied on the participant's brain. (4) Release of MEPs. MEP measured by EMG electrodes.

After obtaining the individual reaction time, the task consisted of four conditions with 10 trials each. In the first condition (rest), the participant was advised not to react to the reaction test. The stimulus of the reaction task was presented and after 80% of the individual RT paired pulses were applied and MEPs were recorded (e.g., if the individual RT was 500 ms, TMS pulses were applied at 400 ms after the stimulus’ release during the reaction task). In addition to this resting condition, three conditions required the participant to actually react to the test and perform a mouse click. TMS pulses were applied at three different time intervals relative to the individual RT. As such, in the second condition after 40%, in the third condition after 60%, and in the fourth condition after 80% of the individual RT, respectively (see Figures 1, 2). This protocol was adapted from Hummel et al. who stimulated at the time points 63%, 75%, 87%, and 97% of RT (on average) in patients with chronic stroke (40). We chose to alter this paradigm as we expected patients with SCI to have a higher excitability (15–18) and with this an earlier activation in preparation of movement.

### 2.4 Data analysis

#### 2.4.1 MEP amplitude height

MEP peak-to-peak amplitudes were marked manually in the measured EMG and the 10 repetitions of each condition were averaged for each participant. Generally, we expected the amplitude of the MEP to rise from 40% to 60% and to 80% as the time of measurement approached the actual movement. However, for some subjects the 80% value was smaller than the 60% value. This might be owing to a bias in time point of stimulation in regard to RT. Despite the training phase of the reaction test, participants decreased their RT during the trials. For this, data of 80% of RT might have not represented movement preparation anymore. Therefore, we decided to focus on the 60%/40% ratio as the outcome. We put our focus on the ratio, not the amplitude height itself, as this might be dependent on further variables like severity and height of the lesion or age and therefore could bias the results. The greater the ratio 60%/40%, the higher the MEP increase toward movement i.e. the stronger the cortical activation.

#### 2.4.2 Statistical analysis

All statistical analyses were conducted using the statistical software package R (41). Due to the small sample size and because ratio data tend to be not well approximated by a normal distribution, we decided to use non-parametric methods. Specifically, for group comparisons we used the ANOVA-type test (for more than two groups) and the Brunner–Munzel test (for two groups) provided by the R package rankFD (42). The methods implemented in rankFD use the so-called relative treatment effect (RTE) as an indicator for the effect size. It describes the probability of a random observation from one sample having a higher outcome value than a random observation from a reference distribution. In the case of two samples, the reference distribution is the other sample. In the case of more than two groups, it is a (weighted) combination of all groups. The RTE ranges from 0 to 1, with a larger RTE in group A than group B indicating that subjects in group A tend to have higher values of the outcome than subjects in group B (43).

## 3 Results

### 3.1 Demographic and clinical data

The 22 included patients (10 women) had an average age of 49.3 years [standard deviation (SD) = 12.7] and varied between an American Spinal Injury Association (ASIA) Score of A to D, complete and incomplete lesion, as well as traumatic and non-traumatic injuries (Table 1). The healthy participants (13 women) had an average age of 46.2 years (SD = 13.9).

### 3.2 Results of the MEP data

Raw data of MEPs and the 60%/40% quotient are displayed in Table 2 for all healthy participants and patients with SCI.

**Table 2:** Average MEP height in mV individually and of each group during rest, at 40%, 60%, and 80%, as well as the quotient of 60%/40%.

HP	Group	Rest	40%	60%	80%	60%/40%
1	Non-sportive	0.76	0.68	1.86	0.96	2.74
2	Non-sportive	0.06	2.63	4.87	3.38	1.85
3	Non-sportive	0.12	0.56	0.95	1.80	1.70
4	Non-sportive	0.35	0.47	2.99	1.59	6.36
5	Non-sportive	0.16	0.82	1.23	2.55	1.50
6	Non-sportive	0.29	1.44	1.53	1.06	1.07
7	Non-sportive	0.19	0.36	0.65	0.50	1.81
8	Non-sportive	0.01	0.30	0.75	0.96	2.50
9	Non-sportive	0.22	0.43	0.14	1.68	0.33
10	Non-sportive	0.22	0.61	0.41	0.14	0.67
11	Non-sportive	0.04	2.06	4.91	3.10	2.39
12	Non-sportive	0.00	0.29	0.35	1.49	1.21
13	Sportive	0.00	0.87	1.20	0.41	1.38
14	Sportive	0.09	1.15	3.43	5.24	2.98
15	Sportive	0.73	0.66	4.22	4.15	6.39
16	Sportive	0.50	1.97	5.77	8.15	2.93
17	Sportive	1.34	2.51	4.47	3.82	1.78
18	Sportive	0.29	1.38	2.19	1.34	1.59
19	Sportive	0.46	2.65	2.19	6.77	0.83
20	Sportive	0.06	1.33	4.00	9.08	3.01
21	Sportive	0.00	0.33	0.63	1.54	1.91
22	Sportive	0.50	2.64	4.72	4.54	1.79
23	Sportive	3.83	3.61	6.15	10.14	1.70
24	Sportive	0.05	0.69	0.45	1.03	0.65
P	Group	Rest	40%	60%	80%	60%/40%
1	Non-sportive	1.92	0.80	4.53	5.21	5.67
2	Non-sportive	0.00	1.22	0.87	0.21	0.71
3	Non-sportive	0.10	0.56	0.50	0.50	0.89
4	Non-sportive	0.04	0.13	0.23	0.12	1.69
5	Non-sportive	0.07	0.14	0.92	1.82	6.57
6	Non-sportive	0.31	0.53	2.32	3.00	4.38
7	Non-sportive	0.24	0.33	0.70	0.87	2.13
8	Non-sportive	0.04	0.07	0.14	0.34	2.00
9	Non-sportive	1.32	1.04	1.49	3.54	1.43
10	Sportive	0.14	0.10	0.71	1.43	7.10
11	Sportive	0.56	0.62	1.65	2.12	2.66
12	Sportive	0.42	0.94	1.65	1.42	1.76
13	Sportive	0.00	0.12	0.57	1.35	4.64
14	Sportive	0.19	0.07	0.69	3.63	10.35
15	Sportive	1.12	2.89	6.14	5.85	2.12
16	Dancer	1.21	1.02	1.54	1.59	1.51
17	Dancer	0.58	6.62	7.27	8.33	1.10
18	Dancer	0.34	3.89	6.30	4.83	1.62
19	Dancer	0.24	0.39	2.44	2.64	6.27
20	Dancer	0.82	1.46	7.64	11.04	5.24
21	Dancer	0.51	0.62	2.03	1.24	3.27
22	Dancer	0.40	0.21	0.44	0.93	2.10

HP, healthy participants; P, patients.

There are neither significant group differences between the healthy participants and patients (*p* = .082) nor between the patient groups (*p* = .238), see Table 3.

**Table 3:** Statistical results for the group comparisons of the 60%/40% MEP quotient.

Variable	Groups	*N*	Mean (SD)	Median (IQR)	RTE (95% CI)	Test statistic	*p*-value
Patients vs. healthy	Healthy	24	2.13 (1.51)	1.78 (1.34–2.56)	0.35 (0.18–0.52)	T (41.29) = 1.78	0.082
	Patients	22	3.42 (2.52)	2.13 (1.64–5.09)	0.65 (0.48–0.82)		
Sport among patients	Non-sportives	9	2.83 (2.15)	2.00 (1.43–4.38)	0.41 (0.26–0.58)	F (2.00, 18.24) = 1.55	0.238
	Sportive	6	4.77 (3.38)	3.65 (2.26–6.48)	0.65 (0.48–0.79)		
	Dancers	7	3.01 (2.01)	2.10 (1.56–4.26)	0.44 (0.28–0.61)		

IQR, interquartile range; CI, confidence interval.

**Figure 3:**
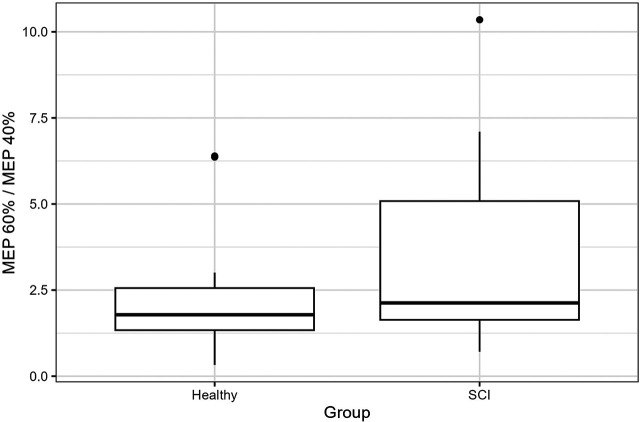
The boxes extend from the first to the third quartiles, with the thick black line presenting the median of the 60%/40% quotient of the healthy participants and patients with SCI.

**Figure 4:**
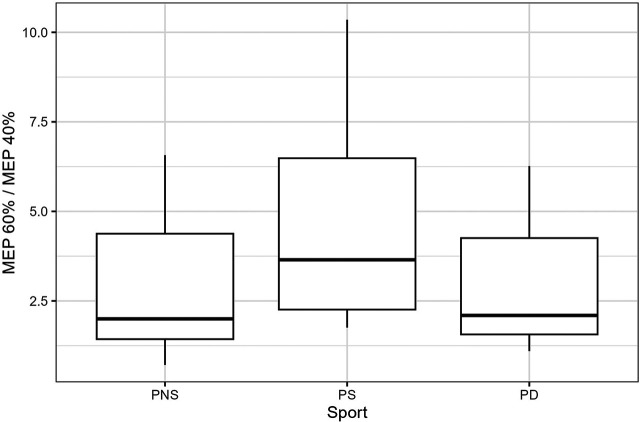
The boxes extend from the first to the third quartiles, with the thick black line presenting the median of the 60%/40% quotient of the patient groups. PNS, Patients non-sportive; PS, Patients sportive; PD, Patients dancer.

Even though not significant, the results show a higher quotient 60%/40% in the patient group compared with the healthy group (Figure 3), while sportive patients show a higher quotient than the dancers and non-sportive patients (Figure 4).

## 4 Discussion

We hypothesized a reduced SICI preceding movement in patients with SCI compared with healthy participants; hence, less increase of cortical excitability due to a constant hyper excitability that is induced by inhibited afferent sensory input (9). We also expected differences in SICI preceding movement between the different patient groups, which were hypothesized to be elicited by sports-specific neuroplasticity. We did not find significant differences in increase of cortical excitability toward movement between the healthy control group and the patients. We consider this is associated with the localization of the stimulation we chose (the motor cortex representing the FDI), which might be affected only to a small extent in some patients. Roy et al. showed that SICI is reduced in patients with incomplete SCI compared with healthy participants. However, they also found larger SICI in the FDI muscle of the hand than in the ankle flexor in patients (17). This might be due to a higher influence of the lesion on the lower extremities than on the higher extremities in the study cohort. In our experiment, the hot spot of the FDI was stimulated. Yet, only 4 of the 22 patients investigated in our study had a lesion higher than the second thoracic level that directly influences the upper extremities. We assume that the results are influenced by a small impact of the lesion on the tested cortical area. Future studies should focus on the cortical excitability of the lower extremities, even though this happens to be challenging via TMS due to the difficult accessibility of this area on the cortex.

Our results did not show significant differences of increased cortical excitability toward movement among the patient groups. These results do not support our hypothesis as we expected neural plasticity to be higher in the sportive groups due to increased BDNF levels (27). Even though aerobic and anaerobic exercises provoke increased BDNF release, aerobic exercise seems to have a higher impact on the brain (44). Aerobic threshold might be higher in patients conducting endurance sports like marathon driving and hand-biking than in wheelchair dancers. According to studies on ballet and modern dancers, training and dance performance demand only little aerobic fitness possibly due to fast and short movements and intermissions in class (45–47). Yet, this assumption could not be supported in our results. We suggest this might be because of the low statistical power owing to the small sample sizes of the subgroups.

However, what is the consequence of an increase of neuroplasticity in patients with SCI? Does it provoke functional changes of the brain that improve the sensorimotor output? According to the hypothesis of negative maladaptive neuroplasticity (48), signals modified after deafferentation can cause a propagation of sensorimotor areas; in the case of paraplegic patients, from the cortex of the upper extremities to the cortex of the lower extremities. Hence, the cortical areas that are affected by the SCI (lower extremities) change their function and are “taken over” by the neighboring areas not affected by the SCI (upper extremities). We consider that activating cortical plasticity, while mobilizing muscles of the upper extremities and neglecting the lower extremities, which is largely the case in wheelchair sports might strengthen the impact of negative maladaptive neuroplasticity. Further studies should investigate this effect and the consequences for patients’ therapeutic outcomes.

Altered SICI evidenced by TMS was also shown in patients with psychiatric disorders like depression (49) and schizophrenia (50). Agarwal et al. summarized that SICI might be reduced in Alzheimer's disease, amyotrophic lateral sclerosis, frontotemporal dementia, Huntington's disease, multiple system atrophy, progressive supranuclear palsy, and Parkinson's disease (51). It is crucial to investigate how different sports influence cortical excitability in these conditions. This might improve therapeutic methods based on exercise on a more individual level.

### 4.1 Limitations

Due to the limited exclusion criteria, a wide range of clinical patterns could be observed in the patients. The patient group was not large enough to be split by age or lesion characteristics, which puts our comparison between the subgroups at risk of being biased by these factors. Every patient showed an individual combination of impairments along with their injury outcome. We did not exclude patients that took medication influencing cortical excitability. The participants were recruited from a real-world patient pool; hence, they were possibly treated with psychotropic drugs. In addition, most patients of the sport groups did not exclusively practice one specific sport alone but were also engaged in other sportive activities. The other sports group contained different kinds of sports that require alternative movement patterns. The examination conditions were not tested in randomized order; hence, a habituation or fatigue effect throughout the experiment cannot be excluded. Neither in the healthy participants nor in the patients was handedness taken into consideration. In addition, examiner-related errors in manual MEP amplitude calculation could have influenced the quality of the data. It should be kept in mind that MEPs reflect not only the isolated cortical activity, but also the processing of signals to the spinal cord, the moto neurons, and the functionality of the target muscle. As this system is disturbed in patients with SCI, the sum of pathological alterations and their consequences can hardly be predicted or taken into account when interpreting MEP amplitudes. Hence, instead of interpreting the individual MEP height, we focused on the ratio between amplitude heights during 40% and 60% of RT. This approach lowers the impact of this limitation.

### 4.2 Future directions

Future studies should include a greater sample size and a more homogenous group of patients with SCI. It is of high relevance for the outcome of a study to include patients with comparable injuries (i.e., injury height, complete/incomplete lesion, traumatic/non-traumatic) especially when investigating the sensorimotor system. It should be considered to not only test SICI but also intracortical facilitation (ISI = 10 ms), MEP latencies, stimulus–response curves, and their correlation with the reaction time. This might reveal deeper insight into inhibitory and excitatory cortical processes in patients with SCI. In addition, cortical excitability should be correlated with cognitive impairment and mood disorders in patients with SCI and other neurological diseases. This might help to uncover the functional impact that altered cortical excitability has on the patient's health. We did not distinguish between beginners and advanced athletes, neither how intensely and for how many years the exercise was conducted, nor did we consider the amount of exercise done before the injury. This might as well have had an influence on the cortical connectivity and should be controlled for in further studies.

In summary, our hypothesis addressing the cortical excitability toward movement between healthy participants and patients with SCI, as well as between patients conducting different sports, could not be supported. The small sample sizes of the subgroups might be the reason for a lack of significantly different effects; thus, this study suffers from low statistical power. Yet, the trending effect warrants further investigation of the impact of different sports on the neuroplasticity in patients with SCI, while the lower extremities should be brought into focus. This might shed light on the correlation between wheelchair sports and maladaptive neuroplasticity in the lower extremities of patients with SCI.

## 5 Key Summary Points

• Cortical structures are highly plastic and adjustable as observed by previous studies on animals and humans after deafferentation.
• We hypothesize a reduced SICI preceding movement in patients compared with healthy participants. In addition, we expect that exercise-induced neuroplasticity activities can modulate intracortical inhibition during movement preparation in patients with SCI.
• We could not establish statistically significant differences between the examined groups.
• A non-significant trend indicates toward the greatest increase of cortical excitability in patients in the sports group and the lowest increase of cortical excitability in the non-sportive group.
• The small sample sizes limit the statistical power of the study, but the trending effect warrants further investigation of the impacts of different sports on the neuroplasticity in patients with SCI.

## Data Availability

The data supporting the conclusions of this article are available upon request from the corresponding author: v.frey@salk.at.
